# Impact of hearing impairment on psychological distress and subjective well-being in older adults

**DOI:** 10.12669/pjms.36.6.2457

**Published:** 2020

**Authors:** Yasmeen Niazi, Bisma Ejaz, Amina Muazzam

**Affiliations:** 1 Yasmeen Niazi, MS Scholar. Department of Applied Psychology, Lahore College for Women University, Lahore, Pakistan; 2 Bisma Ejaz, Assistant Professor, Department of Applied Psychology, Lahore College for Women University, Lahore, Pakistan; 3 Amina Muazzam Tenured Associate Professor, Department of Applied Psychology, Lahore College for Women University, Lahore, Pakistan

**Keywords:** Psychological distress, Subjective well-being, Hearing impairment

## Abstract

**Objective:**

The main objective of this study was to explore the impact of hearing impairment on psychological distress and subjective well-being in older adults with hearing impairment.

**Methods:**

The study with cross sectional research design was conducted in three public sector hospitals of Lahore, from February 2017 to June 2017. Participants of the study were adults aged 50-90 years and with hearing impairment, selected through non-probability sampling technique. Demographic Information sheet, Kessler psychological distress scale by Kessler, Mroczek. in 1992 and Satisfaction with life scale by Diener, Emmons, Larsen, Griffin in 1985 were used for data collection. SPSS 21 was used to analyze the data.

**Results:**

There were 200 participants with age ranged from 53 to 89 years (M= 65.92, SD= 9.70). Of the total, 100 (50%) subjects were men and 100 (50%) were women. Significant gender differences were found in psychological distress, with men reflecting more symptoms of psychological distress (p<0.01), whereas non-significant gender differences were found in case of subjective well-being (p>0.05). Moreover, psychological distress was observed as a predictor of subjective well-being (*p*<0.01). One-way analysis of variance revealed insignificant differences of psychological distress and subjective well-being across three levels of hearing impairment.

**Conclusion:**

Early diagnosis and rehabilitation of age-related hearing loss improves the overall quality of life of older adults living with hearing impairment.

## INTRODUCTION

Hearing impairment is described as the partial or total loss of hearing ability. On the basis of the severity of the problem hearing impairment can be classified as mild, moderate, severe and profound. Age related hearing impairment is a very common disorder in older adulthood. It is considered as third most prevalent chronic health condition in people over 65 years of age.^1^ Hearing impairment in old age typically stems from some pathological changes inside the ear which are related to aging, it may lead to the impairment of low to high frequency hearing. This problem comes up with greater difficulty in listening process that significantly impairs person’s ability to communicate with others, which in turn is a serious concern related to the mental health of individual.^2^ Hearing impairment in adults is specified by threshold greater than 40 dBHL in better hearing ear. There is no proven treatment for the age related hearing loss, the only way to help people with hearing loss is the use of assistive techniques (hearing aids and cochlear implants) that can increase the threshold to a significant level but do not return hearing to normal.^3^

Hearing impairment in aging is substantially associated with disability, increased risk of incident morbidity, poor self-perceived health,^4^ poor psychological well-being, low levels of self-efficacy and happiness.^5^ The problem is also proved to be linked with anxiety, cognitive decline and lower health related quality of life.^6^ Aforementioned symptoms and their morbidity were found conceivably related to the severity of hearing impairment itself.^7^

Psychological distress is defined as the condition of emotional disturbance with feelings of anxiety (e.g., feeling restless and tense), depression (e. g., feeling hopeless and loss of interest). People may experience psychological distress while coping with the stressful, disturbing or harmful circumstances in their daily life.^8^ While studying the psychological distress in hearing impaired older adults it was reported that they were more vulnerable to mental health problems like depression, decreased well-being, emotional sensitivity and aggression.^9^ Hearing loss can prevent people from being exposed to socially challenging circumstances resulting in isolation which significantly impinges upon their quality of life and mental health.^10^ Perceived social disability has been proved to serve as a mediator between hearing impairment and psychological distress including aggression.^11^ It has been reported that adults with hearing loss tend to exhibit more symptoms of depression, anxiety, psychological distress, and emotional sensitivity as compared to people with normal hearing.^12^

Subjective well-being is how a person evaluates his/her life cognitively and emotionally as a whole, it basically includes cognitive judgments concerning life satisfaction and emotional reactions to life events that may be positive or negative.^13^ Hearing loss poses a serious risk to the mental well-being and overall quality of life in older adults. People may experience diminished self-esteem that in effect leads to the poor mental health and decline in psychological well-being.^14^ In a study intended to find out the impact of hearing loss on cognitive and emotional domains specifically, most of the people described that hearing impairment has a significantly negative connotation and it accounts for the poor well-being in general.^15^ It is evident from the literature that higher well-being was significantly linked to decreased mental distress and social isolation in adult population with hearing impairment. The study provides a clear evidence that rehabilitation techniques and proper intervention plan not only have remarkable effect on well-being of people with hearing impairment but it also reduces the psychological distress to a greater extent.^16^

Current study was aimed to gauge the impact of age related hearing impairment on psychological distress and subjective well-being in older adults. Gender differences in psychological distress and subjective well-being were studied. Additionally, predictive relationship between psychological distress and subjective well-being and difference in both variables were investigated.

## METHODS

The cross sectional study was conducted in Lahore, from February 2017 to June 2017. The study sample comprised of 200 older adults 53 to 89 years old with hearing impairment, among the participants 100 (50%) were men and 100 (50%) were women. Non-probability purposive sampling technique was employed to select the sample. The clinical diagnosis of hearing impairment was carried by the physicians using audiometry. People with hearing impairment but less than 50 years of age were excluded from the study.

A brief demographic form along with Kessler Psychological Distress Scale (K10) by Kessler and Satisfaction with Life scale (SWLS) developed by Diener and his colleagues, was used for data collection. Kessler Psychological Distress Scale (K10), was used to measure the mental condition of the participants. Scale consisted of 10 items graded on likert scale of five points (1 = none of the time to 5 = all of the time). K10 was proved to be a better predictor of non specific psychological distress, anxiety and depression and is widely used as a global measure of psychological distress. The scale has good reliability, ranges from 0.89 to 0.91.^17^

Subjective well-being of the participants was assessed by the Satisfaction with Life Scale (SWLS). This is a short form 5 item scale, responses were rated on 7 point likert scale (1 = strongly agree to 7 = strongly disagree). It is widely used scale for measuring life satisfaction having good psychometric properties. The reliability of scale was reported by authors as 0.87.^18^

Prior to data collection informed consent was obtained from the participants after elaborating the purpose of the study, they were assured about the confidentiality of their personal information. Urdu versions of Kessler Psychological Distress Scale (K10) and Satisfaction with Life Scale (SWLS) were administered to collect data about study variables. Personal demographics of patients were obtained on separate sheet prepared to collect information regarding age, gender, marital status, family system, socio-economic status and necessary details about their problem. SPSS 21 version was used for data analysis. Analysis of the basic variables was done by using descriptive statistics. Gender differences among two study variables were studied by using independent sample t-test. Linear regression analysis was used to find out whether psychological distress is a predictor of subjective well-being. One-way analysis of variance was used to find out if there are any differences among psychological distress and subjective wellbeing among different levels of hearing impairment in older adults. Ethical clearance was obtained from the ethical and research committee of the Lahore College for Women University, Lahore with IRB number 138 and issue date of 13-01-20.

## RESULTS

Of the 200 participants 100 (50%) subjects were men and 100 (50%) were women. Overall mean age was 65.92±9.7. Majority 138(69%) participants were living in nuclear family system. Most of the participants 139(69.5%) were married, 123(61.5%) belonged to middle class. Education statistics showed that 105(52.5%) participants were educated and 95(47.5%) were uneducated. Majority 101(50%) were having moderate hearing disability, 71(33.5%) with mild, 22(11%) with severe and 6(3%) were dealing with profound hearing impairment.

Gender differences in psychological distress and subjective well-being in both genders were studied. Psychological distress was reported to be higher in males (p<0.05) as compared to females. But no gender differences were found in subjective well-being (p>0.05) (Table-I).

**Table-I:** Independent sample t-test comprising gender differences in PD and SWB among adults with hearing impairment (n=200).

Variables	Male (n=100)		Female (n=100)		df	t	p	Cohen’s d

	M	SD	M	SD				
PD	33.05	5.19	31.62	3.54	198	2.27	.02*	.32
SWB	18.90	4.27	18.86	4.05	198	.068	.94	.01

PD= Psychological distress, SWB= Subjective well-being

* p<0.05

Linear regression analysis was used to find out the predictive relationship between psychological distress and subjective well-being in adults with hearing impairment. Results indicate that psychological distress was significant predictor of subjective well-being (p<0.01) (Table-II).

**Table-II:** Linear Regression analysis Predicting Subjective Well Being from Psychological Distress (n=200).

Predictor Variable	B	SE	Β	T	P
Psychological Distress	0.17	.06	.18	2.67	0.00
R	0.18				
R^2^	0.03				
F	7.13				

***Note:*** Criterion Variable: Subjective Well-being, p<0.01

One-way analysis of variance was employed to test if there are any differences in perceived psychological distress and subjective well-being among different levels of hearing impairment. Results revealed that there were no significant differences found for both variables in different levels of hearing impairment (p>0.05) (Table-III).

**Table-III:** Summary of one-way analysis of variance (ANOVA).

Variables	Groups	SS	Df	MS	F	P
Psychological Distress	Between Group	136.49	3	45.49	2.29	.07
	Within Group	3880.61	196	19.79		
	Total	4016.55	196			
Subjective Well-being	Between Group	57.49	3	19.16	1.11	.34
	Within Group	3381.62	196	17.25		
	Total	3439.12	196			

***Note:*** SS=sum of squares; df= degree of freedom; MS= mean squares. p>0.05.

## DISCUSSIONS

Hearing impairment is the most common sensory disability in older adult. Review of the previous literature suggests that hearing impairment is negatively associated with the mental health and the quality of life of adults.^19^ In spite of importance of hearing in our life, hearing loss and especially age related hearing loss is often unrecognized and untreated issue. Even people with hearing impairment often do not consider it as a serious problem, as in many societies there is a misconception that age related hearing impairment is the part of normal aging process rather than an unusual disability. As a result of it people tend to neglect the issue and do not take any serious and timely actions to seek the proper treatment which may help stop the progression of problem.

Gender differences in subjective well-being and psychological distress in adults with hearing impairment were explored in the study. No gender differences were found in case of subjective well-being, but for psychological distress men scored significantly higher as compared to women. In old age most of the people consider age related hearing impairment as the part of normal aging process. That is the main reason of not having a major impact of the problem on their subjective well-being. Current findings are in line with the previous literature, where hearing loss was found to be associated with psychological distress in both men and women, but slightly more depressive symptoms were observed in men than it may affect women. It may be due to the reason that men experience an additional source of psychological distress due to the fact that they usually face the societal pressure of being the bread-winner for the family, and the disability hamper their daily life functioning to a greater extent.^20^ Gender based association of depression and cognitive decline was studied, indicating that having both visual and hearing disability was be linked with more cognitive decline leading to higher level of depression in men, as compared to women.^21^ In a study aimed to investigate the association among hearing impairment and mental health in older adults, it was suggested that hearing loss was highly correlated to depressive symptoms in male population than females.^22^ Significantly higher association among hearing impairment and depression was observed in men at their 40s to 50s as compared to those in their 60s. But no significant gender differences were found in both age groups in case of depression related to hearing impairment.^23^ The social acceptance of older adults with hearing impairment can be factor that lead to less depressive symptoms in older population as compared to young people.

Results of the study revealed that psychological distress is a predictor of subjective well-being among older adults with hearing impairment. These findings correspond with the existing literature suggesting that hearing loss actually cuts off the person facing problem form other people of society. People facing problems in communication with others may experience withdrawal from social interactions, feeling of being excluded, poor quality of life and loneliness that lead to the feeling of depression and having higher rate of psychological distress.^12^ People with hearing impairment are at more risk related to maladjustment in social situations, they are susceptible to experience additional disability by having difficulty in facing environmental hazardous. All these factors contribute to the decreased social health that has a significant negative influence on the mental health and well-being of individuals facing the problem.^10^

A study intended to explore the quality of life of people having hearing loss proved that hearing impairment was positively associated with psychological distress, where people experiencing more psychological distress tend to have decreased overall quality of life.^18^ Loneliness and depressive symptoms were also proved to be the significant predictors of poor physical and mental health related quality of life of people with hearing loss.^14^ Role of social exclusion and other mental health demographics in suicidal ideation were studied, it was evident that the psychological distress, age and severity of hearing loss were the risk factors or predictors of suicidal ideation while psychological well-being was indicated as negative predictor of suicidal ideation.^16^ People suffering from hearing loss experience depression which is often linked to substantial comorbid impairment that can negatively influence the daily life responsibilities. This situation leads to the cognitive decline, anxiety and low mental well-being.^9^

Subjective wellbeing and psychological distress in older adults dealing with different levels of hearing impairment was studied. Present study provided evidence that no significant differences were found in both variables among varying levels of hearing impairment. It is evident from the literature that problem in high or medium frequency hearing has nearly no impact on mental health if low frequency hearing was not affected.^24^ It is well documented that if the problems related to hearing loss are understood properly and new strategies helpful for the improvement of different levels of hearing impairment are learned, this leads to improved self-perception of quality of life. Hearing loss can have negative influence on people’s daily life activities, social activities and emotional state but these individuals usually do not develop the symptoms of clinical depression and anxiety if they are provided with proper social support.^25^ On the contrary some studies suggest that individuals with moderate and greater hearing impairment have more chances of getting the depressive symptoms as compared to those with mild or no hearing impairment.^5^ This lack of association may be due to some cultural differences that can influence the relationship between hearing impairment and mental health in older adults, and also due to the fewer participants in severe and profound hearing impairment groups.

### Implications

This study will provide an important insight for health care practitioners to introduce new plans and policies to figure out the issue at initial level and to prevent mental health problems and improve the well-being of adult population with hearing impairment. As the age related hearing impairment is slightly unnoticeable disability, so it can be easily neglected by the people, especially if they are having any other major health related problems. This situation necessitate the formulation of effective framework for the timely identification of the problem and to ensure the availability of proper hearing aids and other assistive techniques to the people already having or at risk of hearing loss. It is recommended that professionals working with this population should carry out screening for psychological distress along with the physical monitoring of hearing loss. Social support is an important aspect regarding the management and rehabilitation of older adults with hearing impairment.

### Limitations of the study

Sample of research comprised of participants from few public sector hospitals of only one city, it was mainly due to lack of resources. Psychological impact of the hearing impairment may vary in different areas or countries. To resolve the concerns related to external validity studies should include multiple public and private sector institutes form different areas of Pakistan. Future researches are needed to elucidate the relationship of hearing impairment and mental health involving more personal and environmental factors more importantly.

## CONCLUSION

Gender appear to be a variant in Psychological distress reflecting high distress in men with the said condition however for the subjective well-being there were no gender differences observed. Moreover, psychological distress was found to be a predictor of subjective well-being in adults with hearing impairment and no significant differences in psychological distress and subjective well-being were found in different levels of hearing impairment.

### Authors’ Contribution:

**YN:** Conceived, designed and did statistical analysis & editing of manuscript. She is also responsible and accountable for the accuracy or integrity of the work.

**BE:** Supervised research.

**AM:** Did review and final approval of manuscript.
